# Observations on Muscular Contraction

**Published:** 1809-06

**Authors:** G. Barzellotti

**Affiliations:** Professor of Medicine and Surgery in the Royal University of Sienna


### *Observations on* MUSCULAR CONTRACTION, *by* G. BARZELLOTTI, *Professor of Medicine and Surgery in the Royal University of Sienna. Translated from the Italian.*

Jus anceps novi, causas defendere possum. HORAT. Sat. v. Lib. ii.

MR. CARLISLE'S Memoir on muscular motion, recently published in the English and some foreign Journals, makes no mention whatever of various facts relative to this important subject, which had been previously published in England, and also by myself, upwards of ten years ago. I do not mean to accuse Mr. Carlisle of illiberality, but I wish it to be known that in 1796, I published at Sienna an examination of some modern theories upon the cause of muscular contraction, which afterwards appeared in the Journal of Venice, the *Opuscoli Scelti* of Milan, and the Journal of Naples for the same year.* Having made these preliminary observations in justice to myself and others, I shall proceed to examine Mr. Carlisle's memoir. I shall commence by a detail of the phenomena, afterwards pass to their causes, and then communicate my own experiments.

The animal motions which proceed from muscular motion, have been latterly interesting subjects of inquiry among physiologists. Although before the days of Haller, the contracting faculties of the muscles had been discovered, this property had rather been regarded as an effect, than as the proximate cause of motion. The cause of muscular irritability was sought for in the animal spirits, in fermentations, in air, or in æther. Haller, by studying this contraction in all its ramifications, found that it was owing to a property inherent in muscular organization; he fixed its laws, and assigned as the proximate cause, the principle which he called *irritability*. From that moment the ideas of men of science were concentrated on the subject, and the old theories were abandoned. The analytical spirit of the eighteenth century, however, was not contented with this discovery: new systems were sought for, and they were founded on ascertained facts.

The influence of the blood upon muscular contraction had been more or less appreciated previous to Haller by eminent anatomists and physiologists. They had ascer-

* It should be here remarked, that Mr. Carlisle, at the beginning of his Paper, notices his intention to decline any references, whilst he acknowledges his obligations to different authors.——ED.

tained, that without this fluid, life could no more be supported in the muscles than in the other parts of the body. Some concluded that it was the cause of contraction. But if the muscle was paralyzed by placing an obstacle in the way of the circulation of the blood, it follows that any hindrance to the return of this fluid would have a similar effect, and that when the circulation of the blood takes place freely in the muscle, it would be sufficient to tie a nerve in order to paralyze it. Hence it would result that irritability did not depend upon the influence of the blood, but upon the nerves. Haller regarded these organs as stimulant in voluntary motions, and on that account an occasional cause of contraction; but he attributed the stimulant property to the blood in involuntary motions, as for example, in the motion of the heart; he thought that the blood conveyed its heat into all the muscles, and supplied the means of supporting their organization, and that the nerves augmented the *vitality* even where muscular motion is involuntary, such as the heart and intestines.

Prokasca of Prague, after having very carefully examined the most subtle structure of the muscular fibres, and remarking that they were strewed over with and surrounded by an infinity of vessels, thought for the first time, that the blood in proceeding to these vessels dilated those which were situated transversely, and contracted those which were in the direction opposite to it, effects which according to him were sufficient to explain the phenomenon in question. Hence it follows, that the blood must arrive at the muscle in greater quantity at the moment of its contraction; and for this reason, the muscle should at the same moment increase in volume*. Glisson in England, at the time when the theory of animal spirits was most in vogue, also thought that at the moment when these spirits arrived at the muscle, the latter should augment in volume; † and some time afterwards Blane, in order to support one of his favorite systems, also asserted that the volume of the muscles became more considerable at the moment of contraction‡.

The same opinion had been adopted from the time of Haller; and it had been observed, that the muscles were more highly coloured at the moment of contraction, which gave probability to the supposition that the blood flowed in greater quantity. But Glisson and Blane were the first who supported their theory by the experiments I am about to mention. Glisson

* De musculorum actione.

† Tractatus de ventriculo et intestinis. Cap. 8, p. 191.

‡ On the cause of muscular contraction by Dr. G. Blane.

Glisson prepared a glass tube large enough to hold a man's arm; at the extremity, towards the shoulder, the tube had a branch, which rose perpendicularly, and terminated in the form of a small funnel. He introduced the arm of a man into the large branch up to the shoulder, and closed up all the joinings with mastic, in order that the tube might contain water, with which it was filled by the extremity of the branch terminating in a funnel. The man was then directed to contract the muscles of his arm, and afterwards to relax them, while the motion of the water in the small branch was carefully observed. Glisson says he remarked, that the water fell in the small branch at the moment of the contraction, and rose at the period of relaxation. He asserted, that not only the volume of the muscle was not increased at the time of its contraction, but that on the contrary it was diminished: hence his experiment was not more favourable to the partisans of the system of animal spirits, than it was afterwards to those who maintained the theory of the accumulation of the blood.

Blane, with different views, and with more success, tried to measure the volume of the muscle in the two opposite states of contraction and relaxation. For this purpose he took the posterior half of an eel: he introduced it into a flask full of water, the neck of which he contracted by means of the blow pipe, until it was not wider than the tube of a thermometer. He took care that the water should rise to a certain point in the little neck, thus converted into a small tube; he then introduced a fine iron wire, with which he pricked the tail of the eel. Very strong contractions were immediately produced, and he observed the effeet which it had on the level of the water; but he never perceived any alteration in this level, although he varied the experiment by dividing the eel into a greater number of parts. It seems from the experiments of this author, that the muscle neither contracts nor diminishes in volume at the moment of its contraction. My opinion upon these two experiments is, that the first was made in an unsatisfactory manner, and upon a part of the body which is not easily enough enclosed in a tube to observe the minutest changes: we can only regard it as a curious fact, from which it results, that the relative weight or size of a muscle, in two opposite states, with respect to its action, are not sensibly different. This is the opinion of Manget, and other respectable authorities*.

 The

* Theatr. Anat.

The second experiment, although ingenious, could not I think have been very exact; for the motion of the iron wire when the eel was touched with it, should of itself occasion some variations in the level of the liquid. A method was still wanting which should admit of our observing easily, and with precision, the two states of the irritated muscle, without the inconveniences attending the above experiments: this is what, after long search, seems to be I think at last attained.

I employed a glass vessel of a conical form, from the lower part of which there rose laterally a tube as slender as that of a thermometer, and which went some inches beyond the truncated summit of the vessel, which was filled with water. I placed a piece of silver at the bottom, and introduced the interior half of a frog, the crural nerve of which was armed with tin, as directed by Galvani. This nerve thus armed, a little beyond the articulation, was directed towards the bottom of the vessel; it was brought close to the piece of silver, and supported by a small brass hook, to which the leg was attached: the wire of the hook passed through a stopper of soft wax, which entirely closed the aperture of the vessel. On pushing down the wire until the armed part of the nerve touched the piece of silver, I had always sufficient time to examine attentively the height of the fluid in the tube of observation; and when I excited the muscular contraction, I could perceive if any change took place in the level, as also if the level changed, when relaxation succeeded contractious by alternatives, during a sufficient length of time, without having occasion to repeat the stimulants.

The level of the water remained invariable in the opposite states, and every time the experiment was repeated; the discovery of Blane was thus confirmed, as well as the error of Glisson. It is therefore clearly established, that the muscle does not change its volume in the state of contraction or of relaxation, and consequently, it does not receive more blood in the first of these two states than in the second.

Mr. Carlisle in the second part of his ingenious inquiries respecting Muscular Motion, and precisely at the place which contains the facts and experiments in support of his theory, after having determined the influence of temperature upon muscular contraction, endeavours to determine the effect of this contraction upon their volume; and details an experiment which is certainly ingenious, although perhaps not conclusive. He introduced a man's arm into a glass a glass cylinder, so as to make the skin contiguous to the glass at the shoulder: after having filled it with water at the temperature of 97° of Fahrenheit, he luted the arm to the orifice. The cylinder was then closed at the other end by a plate of ground glass, and placed in communication with a tube which issued parallel to the axis of the cylinder, and acted as an index for announcing the smallest variations of the fluid. No air remained in the apparatus, and upon the slightest contractions of the hand or fore arm, the water rose rapidly into the tube, in which it formed oscillations of six or eight inches, corresponding to the alternations of the muscular action. Here it seems Mr. Carlisle concluded, that the muscle increases in volume when contracted, and diminishes when relaxed. He afterwards says, however, that the quantity of blood with which the muscle is impregnated, is less in its state of contraction than in its state of rest; it seems therefore, that he does not admit this augmentation of volume as the effect of increased quantity of blood, since he adds, that he considers the action of the blood-vessels as less during the contraction of the muscle than during its relaxation. A French physiologist,* taking Glisson's experiment as correct, has admitted an apparent augmentation of volume in the muscle during its contraction, and a real diminution at the same time, a diminution which partly proceeds from the depression of the fatty texture compressed within the muscular interstices, and partly from the irritable parts receiving less blood. His opinion, if it is not more correct, is at least more consistent; but it is founded upon an inaccurate experiment.

Which of us then has a priority in point of date, and of the accuracy of his experiments? and which of us is best entitled to belief? Is it Glisson and his partisans, who admit the diminution of volume in the muscle at the instant of contraction? Is it Blane and myself, who distinctly prove that the volume undergoes no alteration whatever at this instant? Or, last of all, is it Carlisle, who maintains that we may calculate extremely well the augmentation in volume which takes place at the moment of contraction, and the diminution which accompanies the relaxation? I am of opinion, however, that it will be regarded as indubitable, that Carlisle's experiment is the same with that of Glisson, and that the experiment of

 Blane,

* Anselme Richeraud, *Nouveaux Elemens de Physiologie.*

Blane, which differs a little from it, is far more important, and should not be passed over in silence. Still less should mine be neglected, which if it is not new, is not the same with the two former, for it bears the stamp of accuracy, and it never can be called in question, if repeated in the manner which I have described.

Mr. Carlisle may fairly be charged with not having done justice to his countrymen, or he must have been ignorant of their experiments and mine; or rather he seems to have knowingly and willingly appropriated to himself the experiment of Glisson. In neither of these points of view has his conduct been favourable to true philosophy. With respect to myself, I do not think my experiments have ever been heard of by M. Carlisle, and from this motive I publish them through the present channel,* in the hope that they may reach him, and that they will neither displease nor injure him.

Since, according to my experiment, the volume of the muscle does not change when contracted or relaxed, we can no longer conclude with Procaska, that a greater quantity of blood contributes to shorten the muscle; nor that the quantity of blood is less, because the irritability of the fibres closes up the orifice of the blood vessels, as Glisson and Carlisle believed. But as we may imagine that the vessels which accompany the last elementary muscles, are not in the same state with those covered by the interstices and superficial parts of the muscles, and that consequently some are filled with blood in the same ratio that others are emptied; and that it is sufficient the volume of the muscular elements be augmented sufficiently to enlarge the fibres while the volume of the interstices, and of the superficial parts diminishes in such a manner, that the volume of the muscle is the same in every respect during contraction, as it was previously; from all this I say (it results as observed by me and by Richerand several years afterwards) that the experiments of Glisson, Blane, Carlisle, and myself become insufficient, and are susceptible of all the doubts which philosophy suggests, if we cannot support them by new experiments. I do not know whether Glisson and Blane have started this difficulty. I shall briefly state how far I went in order to resolve it.

If the blood, said I, flows to the minutest vessels of the muscle, in order to increase the diameter, and to shorten their

* Bibliotheque Britannique.

their longitudinal axis, then to dilate the transverse fibres, and to produce contraction by increasing the volume, it will be enough to cut these vessels suddenly at the moment of contraction, and to place them in the focus of a microscope, in order to discover the effect in dispute. I performed this experiment under the lens of a microscope made by Adams, and I excited the contraction of the muscle to which the cut fibres belonged, but I perceived no motion of the blood in the apertures. I was afterwards anxious to ascertain by inverse experiments, if the muscle could really be contracted without the blood flowing there in greater quantity. For this purpose, I inserted into a frozen medium pieces of the muscles, and some entire frogs. I was then convinced that the blood was coagulated in the small and large vessels. I then excited the contraction of the muscles by galvanism, which operated in the most efficacious manner. From this I concluded, that my first experiment upon the measure of the volume of the muscle was exact, and that the blood had no influence in the phenomenon of muscular contraction. On continuing my inquiries into the same subject, I exposed some muscles to a greater cold than was necessary for coagulating the blood, and I saw that although the vitality of the muscle was thereby diminished, it was still susceptible of contraction by employing active stimulants, from which I concluded, that muscular action not only had no essential connection with the motion of the blood, but not even with animal heat, or the temperature of the muscle. Of this I was convinced by two distinct experiments. Of two muscles, the one at a common temperature, the other exposed to a freezing mixture, the latter (every other circumstance being the same) had a greater facility in contracting, and preserved this facility for a longer time; a circumstance which I do not think has been hitherto noticed.

After these details, I must call the attention of my readers to the delicate experiments of Mr. Carlisle, as to the effects of temperature upon muscular contraction. He has estimated the effects of the two extremes of temperature, and although he found that at a higher temperature, as well as at a temperature lower than ordinary, the muscles lost a part of their irritability, he has nevertheless admitted, that freezing did not destroy it. After having kept for eight successive hours the lower extremity of a skinned frog at a temperature nearly freezing, he found

 that that after the limbs were frozen, the irritability reappeared completely as before.

I do not wish to extend my observations upon the Memoir of Mr. Carlisle beyond what concerns myself. I am satisfied with informing the public that as long ago as the year 1796, I had most rigorously demonstrated that the muscle is not increased at the moment of its fibres being shortened; that the blood does not flow into the minutest vessels of the muscle in dead animals, when we excite contractions by means of stimulants; that the muscles are contracted when the blood is frozen in their vessels; and, finally, that the force of contraction of the muscles is not diminished when they are deprived of blood; all which, I think, I have completely established by my experiments; in a word, muscular contraction does not directly depend either upon the blood or upon the temperature.

I am anxious that the ingenious Mr. Carlisle should be acquainted with my inquiries into a question, upon which he has thrown so much light, and that he should know how much I esteem him and his labours.

